# A Rare Form of Retinal Dystrophy Caused by Hypomorphic Nonsense Mutations in *CEP290*

**DOI:** 10.3390/genes8080208

**Published:** 2017-08-22

**Authors:** Susanne Roosing, Frans P. M. Cremers, Frans C. C. Riemslag, Marijke N. Zonneveld-Vrieling, Herman E. Talsma, Francoise J. M. Klessens-Godfroy, Anneke I. den Hollander, L. Ingeborgh van den Born

**Affiliations:** 1 Department of Human Genetics, Radboud University Medical Center, 6525 GA Nijmegen, The Netherlands; Susanne.Roosing@radboudumc.nl (S.R.); Frans.Cremers@radboudumc.nl (F.P.M.C.); Marijke.Zonneveld-Vrieling@radboudumc.nl (M.N.Z.-V.); Anneke.denHollander@radboudumc.nl (A.I.d.H.); 2 Donders Institute for Brain, Cognition and Behaviour, 6525 EN Nijmegen, The Netherlands; 3 Bartiméus Institute for the Visually Impaired, 3703 AJ Zeist, The Netherlands; FRiemslag@gmail.nl (F.C.C.R.); HTalsma@bartimeus.nl (H.E.T.); 4 The Rotterdam Eye Hospital, 3011 BH Rotterdam, The Netherlands; 5 Sint Franciscus Gasthuis, Department of Internal Medicine, 3045 PM Rotterdam, The Netherlands; F.Klessens@Franciscus.nl; 6 Department of Ophthalmology, Radboud University Medical Center, 6525 EX Nijmegen, The Netherlands

**Keywords:** oligocone trichromacy, cone dysfunction syndrome, *CEP290*, ciliopathies, long-term follow up

## Abstract

Purpose: To identify the gene defect and to study the clinical characteristics and natural course of disease in a family originally diagnosed with oligocone trichromacy (OT), a rare congenital cone dysfunction syndrome. Methods: Extensive clinical and ophthalmologic assessment was performed on two siblings with OT and long-term follow up data were analyzed. Subsequently, whole exome sequencing (WES) and Sanger sequence analysis of *CEP290* was performed in the two siblings. Additionally, the identified *CEP290* mutations were analyzed in persons with achromatopsia (ACHM) (*n* = 23) and autosomal recessive or isolated cone dystrophy (CD; *n* = 145). Results: In the first decade of life, the siblings were diagnosed with OT based on low visual acuity, photophobia, nystagmus, and absent cone response on electroretinography , but with normal color discrimination. Over time, the phenotype of OT evolved to a progressive degenerative disease without any *CEP290*-associated non-ocular features. In both siblings, two nonsense mutations (c.451C>T; p.(Arg151*) and c.4723A>T; p.(Lys1575*)) in *CEP290* were found. Previously, p.(Arg151*) was demonstrated to induce nonsense-mediated alternative splicing events leading to intact open reading frames of the resulting mRNA products (p.(Leu148_Glu165del) and p.(Leu148_Lys172del)). mRNA analysis for p.(Lys1575*) confirmed a suspected hypomorphic character, as exon 36 skipping was observed in a small fraction of *CEP290* mRNA, resulting in a 36 aa in-frame deletion (p.(Glu1569_Trp1604del)). No additional cases carrying these variants were identified in the ACHM and CD cohorts. Conclusions: Compound heterozygous hypomorphic mutations in *CEP290* may lead to a rare form of cone-dominated retinal dystrophy, a novel phenotype belonging to the *CEP290*-associated spectrum of ciliopathies. These findings provide insight into the effect of *CEP290* mutations on the clinical phenotype.

## 1. Introduction 

Centrosomal protein 290 (CEP290, MIM#610142) encodes a widely expressed centrosomal and ciliary protein of 290 kDa that plays an important role in ciliary trafficking and cilium assembly [1,2]. In the photoreceptors, CEP290 localizes to the connecting cilium, the transitional zone linking the inner and outer segments of rods and cones [1,3,4,5]. To date, over 100 *CEP290* mutations have been identified leading to a spectrum of phenotypes ranging from isolated early-onset retinal dystrophy [6] and Leber congenital amaurosis (LCA, MIM#611755) [4] to more severe syndromes such as Senior Løken syndrome (SLSN, MIM#610189) [7], Joubert syndrome (JBTS, MIM#213300) or Meckel–Gruber syndrome (MGS, MIM#611134 [8]). Hypomorphic *CEP290* mutations are generally associated with non-syndromic forms of LCA, and account for an estimated 15% of all LCA cases in the Caucasian population [4,9,10]. Littink et al. described a family with better preserved central vision; these patients were heterozygous carriers of the most common *CEP290* variant, the deep-intronic c.2991+1655A>G change. This hypomorphic variant generates the splice site for a cryptic exon with a premature stop codon in about 50% of the resulting mRNA products [4]. On the other allele, the c.451C>T (p.(Arg151*)) variant was identified [6], which was found to result in a nonsense-associated altered splicing event leading to an intact open reading frame of the resulting mRNA (p.(Leu148_Glu165del), and p.(Leu148_Lys172del)), possibly explaining the milder phenotype. In two patients of the family, residual rod function was measured on electroretinogram (ERG). 

Here, we report a novel combination of hypomorphic variants in *CEP290* in two siblings initially diagnosed with oligocone trichromacy (OT), whom over time developed a progressive retinal dystrophy. 

The family was first diagnosed by Van Lith, after describing OT as a separate entity [11]. To date, 13 cases of this rare type of cone dysfunction have been described, characterized by non-recordable cone responses on ERG and normal or near normal trichromatic color vision with normal fundi [12,13,14,15,16]. Concomitant signs may include nystagmus and photophobia.

Mutations in *CNGA3* (MIM#600053) [12], *CNGB3* (MIM#605080) [13], *GNAT2* (MIM#139340) [14], and *PDE6C* (MIM#600827) [13] have been detected in four probands with OT, which all encode cone phototransduction pathway proteins [13], suggesting a pathomechanistic overlap with achromatopsia (ACHM). In seven probands with OT, causative variants were not identified in these genes, suggesting further genetic heterogeneity [13,14].

In this report, we present forty years of clinical follow up in two siblings with a rare form of cone dominated retinal dystrophy, carrying compound heterozygous hypomorphic variants in *CEP290*, thereby broadening the spectrum of *CEP290*-associated disease.

## 2. Material and Methods

The research procedures followed the tenets of the Declaration of Helsinki (Seoul) (2008) and were approved by the ethics committee of the Erasmus Medical Center, Rotterdam (ABR-NL34152.078.10). Written informed consent was obtained from the subjects after explanation of the nature of the study. 

### 2.1. Clinical Examination

The first ophthalmic examinations of the subjects date from 1971, at ages 2 and 3. Since then, they were followed regularly and underwent electrophysiology and color vision testing (Hardy Rand Rittler (HRR) Pseudoisochromatic Plates and Farnsworth Panel D-15 Test, saturated and desaturated version) at several occasions. Recent examinations included best corrected visual acuity, biomicroscopy, ophthalmoscopy, fundus photography, color vision testing (Panel D-15), and Goldmann kinetic perimetry. ERG testing was performed according to the extended protocol of the International Society for Clinical Electrophysiology of Vision (ISCEV) [17]. Spectral domain optical coherence tomography (OCT) and fundus autofluorescence (FAF, Spectralis HRA-OCT, Heidelberg, Germany) were carried out as described previously [18], but failed partially due to photophobia and nystagmus. Both individuals were also seen by a specialist for internal diseases to evaluate the presence of extra-ocular features. This included physical examinations and extensive laboratory testing.

### 2.2. Genetic Testing

To determine the genetic defect in the family with two affected siblings, blood samples for molecular genetic analysis were collected from all family members. DNA samples of both affected individuals were analyzed using the GeneChip Genome-Wide Human single nucleotide polymorphism array 5.0 *NspI* (Affymetrix, Santa Clara, CA, USA). Genotypes were called using Genotype Console (Affymetrix) and homozygous regions were calculated with Partek Genomics Suite software (Partek, St. Louis, MO, USA). Subsequently, DNA samples of the affected siblings were analyzed by exome sequencing. The exomes were enriched according to the manufacturer’s protocol using Agilents’ SureSelect Human All Exon v.2 Kit (50 Mb), which contains the exonic sequences of approximately 21,000 genes (Agilent Technologies, Inc., Santa Clara, CA, USA) and next generation sequencing was performed on a SOLiD4 sequencing platform (Life Technologies, Carlsbad, CA, USA). LifeScope software v2.1 (Life Technologies) was used to map color space reads along the hg19 reference genome assembly. The DiBayes algorithm, with high-stringency calling, was used for single-nucleotide variant calling. The small Indel Tool (Life technologies) was used to detect small insertions and deletions.

### 2.3. Sequence Analysis of the CEP290 Gene

The 54 exons and exon–intron boundaries of *CEP290* (GenBank NM_025114) were screened in the DNA of the individuals for the presence of a second mutation as previously described [19]. Sequencing and segregation analysis was performed by Sanger sequencing. Subsequently, a subset of 23 ACHM and 145 cone dystrophy (CD) individuals, mainly from the Netherlands, was specifically analyzed for the occurrence of the c.451C>T and c.4723A>T variants.

### 2.4. Analysis of Hypomorphic Character of the c.4723A>T Variant

To determine the effect of c.4723A>T on the *CEP290* mRNA, RNA was isolated from Epstein-Barr virus (EBV)-transformed lymphocytes of the affected persons and three control individuals according to the manufacturer’s protocol (Qiagen, Venlo, The Netherlands). The lymphocytes of the affected individuals were grown with or without cycloheximide (CHX) to visualize the effect of the mutation and possible degradation of nonsense-containing mRNAs by nonsense-mediated decay (NMD) [20]. Reverse transcription with iScript (Biorad, Veenendaal, The Netherlands) was performed on total RNA (1 µg) to obtain cDNA. RT-PCR experiments were performed using 2.5 µL cDNA with primers in exon 34 and 37 (35 cycles), with a subsequent nested PCR using 0.5 µL of the PCR-product and initial primers (15 cycles), followed by Sanger sequencing.

## 3. Results

### 3.1. Clinical Findings 

The patients were initially examined at the ages of 2 and 3 years, and presented with low visual acuity, severe photophobia and pronounced nystagmus. ERGs were performed under general anesthesia and revealed normal rod responses with absent cone responses. The fact that they could name colors correctly puzzled Van Lith, but they were too young to perform a color vision test. A few years later, they were capable of executing the HRR, and Panel D-15 test correctly, leading to the diagnosis of OT. Remnant cone function was measured on ERG of individual II-2 at the age of 13 with a visual acuity between 20/200 and 20/125, which remained stable over the years.

**Figure 1:**
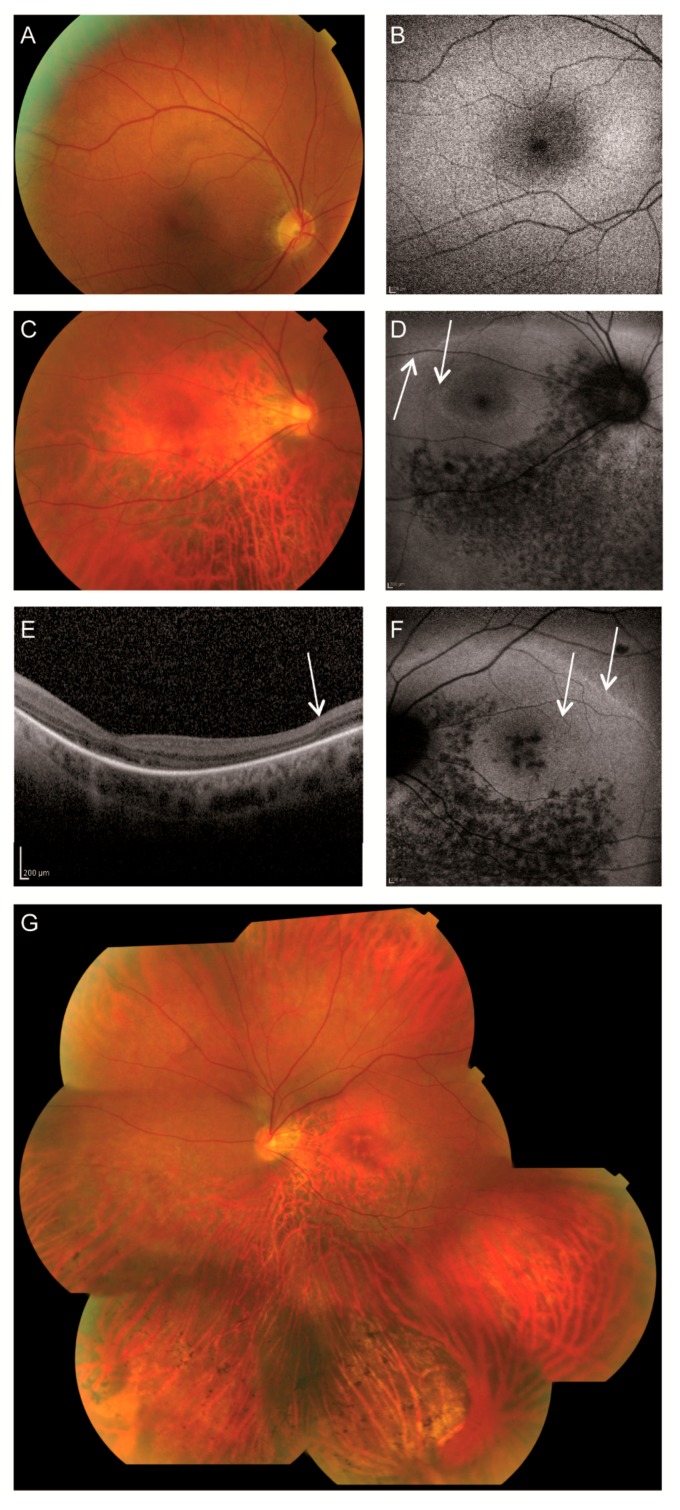
Retinal imaging of two individuals with *CEP290* variants A–B, Clinical characteristics of patient II-1 of family A. (**A**) The fundus photograph showed a pink optic disc and mildly attenuated vessels. The retinal pigment epithelium (RPE) had a coarse-grained aspect in the posterior pole and mid-periphery, and a faintly recognizable foveola reflex with a subtle indication of bull’s eye-like maculopathy. (**B**) Fundus autofluorescence (FAF) revealed a relatively normal appearance. C–G, Clinical characteristics of patient II-2 of family A. (**C**) Fundoscopy displayed a pink, myopic optic disc, mildly attenuated vessels and subtle RPE changes in the macula with a faintly recognizable foveola reflex and thinning of the perimacular RPE. (**D**) Fundus autofluorescence of the right eye revealed a double hyperautofluorescent ring and hypoautofluorescent spots along and inferior to the inferior vascular arcade. (**E**) Optical coherence tomography (OCT) of the left eye displayed no discernible photoreceptor complexes at the macula, but recognizable outer retinal layers at the peripheral part of the scan. (**F**) Fundus autofluorescence of the left eye revealed a double faint hyperautofluorescent ring and hypoautofluorescent spots along and inferior of the inferior vascular arcade as well as in the macula. (**G**) A compilation of fundus photographs of the left eye of II-2 showed a pink and myopic optic disc and mildly attenuated vessels. A faintly recognizable foveola reflex was noted, as well as perifoveal RPE atrophy. Mild RPE changes in the superior quadrants and more pronounced RPE atrophy in inferior quadrants with bone-spicule pigmentations were documented.

At the age of 24, individual II-1 had been tested with heterochromatic red and green checkerboard (60’) reversal (eight reversals per second) visual-evoked potentials (VEPs). A heterochromatic match was found for the green stimulus reduced by 10% transmission absorptive neutral density (1 ND) filter compared to the value found in healthy subjects (unpublished data F.C.C.R.). It thus appeared that the long-wavelength (L) cones were a factor 10 less sensitive than in a healthy situation. Around the age of 27, color vision with the Panel D-15 test was still normal, but both individuals developed more difficulties with pseudoisochromatic plates. ERG rod function was also mildly reduced and perimetry revealed mild visual field defects. At the age of 39, subject II-2 presented with altitudinal visual field defects, while individual II-1 had developed tunnel vision (Figure 1). On fundoscopy, both individuals had degenerative changes mainly in the inferior quadrants consisting of retinal pigment epithelium (RPE) atrophy with bone-spicule pigmentations. In person II-2, the degeneration had progressed towards the macula and small atrophic spots surrounding the fovea were observed in the left eye (Figure 1F). In the last six years, a further deterioration of the visual fields and rod ERGs was observed, whereas visual acuity and color vision were unchanged. The data of the most recent examination are presented in Table 1. Due to photophobia and nystagmus, OCT and FAF failed partially. Interestingly, a double ring sign was observed on the FAF of individual II-2, indicating the transitional zone between present and absent photoreceptors on OCT (Figure 1D,F). A compilation of all fundus photographs of the left eye is shown in Figure 1G. Examinations by a specialist in internal diseases did not reveal any extra-ocular abnormalities that might be associated with the *CEP290* variants.

### 3.2. Genetic Analysis 

**Figure 2:**
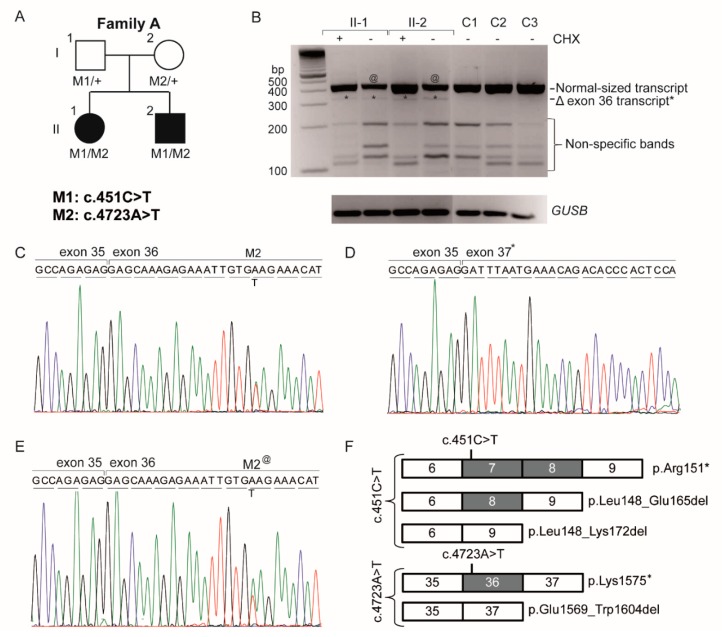
Pedigree and mRNA analysis of individuals with oligocone trichromacy (OT) carrying the heterozygous *CEP290* variants c.4723A>T and c.415C>T. (**A**) The pedigree with OT and the segregation of *CEP290* variants c.415C>T (p.(Arg151*), (M1) and c.4723A>T (p.(Lys1575*), (M2); (**B**) *CEP290* cDNA analysis of the effect of the c.4723A>T variant on splicing of exon 36 in the patient cell lines treated with (+) or without (−) cycloheximide (CHX) to prevent degradation by nonsense-mediated decay (NMD) versus non-treated control cell lines (C1–C3). Agarose gel electrophoresis revealed a major 432 bp product representing the normal-sized transcript in all tested samples. Hence, the difference of band intensity (^@^) between treated and untreated samples in the affected individuals can be explained by the partial effect of NMD on the mRNAs carrying the p.(Arg151*) and p.(Lys1575*) variants. An aberrant product (*) is seen in the affected persons at 324 bp, whereas this product was not found in controls (C1–C3). This product size corresponds to a transcript lacking exon 36. The smaller-sized fragments represent nonspecific products as observed by sequencing. The comparison for input was made by the analysis of the housekeeping gene *GUSB*; (**C**) Sequence analysis of the abundant cDNA product in the CHX treated samples shows the exon transition from 35 to 36 with apparently equal amounts of mRNAs carrying the p.(Lys1575*) mutation and the mRNAs derived from the three c.415C>T alleles (see panel F). (**D**) Sequences of the aberrant product from exon 35 to 37, skipping exon 36. (**E**) Sequence analysis of the CHX untreated normal-sized mRNA product shows a reduced presence of the mutant allele due to partial NMD, visible by a reduced peak size of the mutant T-allele (M2^@^) compared to the wild-type A-allele. (**F**) Schematic representation of the effect of the *CEP290* stopmutations. The c.415C>T variant results in three mRNA variants, one of which carries the truncating mutation and the other two carrying in-frame deletions. The c.4723A>T variant results in two mRNA variants, a major product carrying the p.(Lys1575*) truncating mutation and a minor product carrying an in-frame deletion. Both variants are therefore considered hypomorphic variants.

The analysis uncovered 36,457 variants for individual II-1 and 37,944 variants for individual II-2. A total of 3525 functionally relevant variants, occurring with a frequency of less than 1% in 1302 individuals in an in-house exome database, were shared by both individuals (data not shown). None of the ACHM-associated genes carried pathogenic variants, nor did the other currently known inherited retinal disease genes. Variants were further analyzed when they met our criteria of a frequency <0.5% in the variant databases exome aggregation consortium(ExAC) [21] and 1000 genomes [22], as well as the in-house database (*n* = 29,076 alleles), and we focused on nonsense, frameshift, canonical splice site or missense variants with a PhyloP >2.7. Under the hypothesis of autosomal recessive inheritance, we did not identify compound heterozygous variants (>20% variation) nor any homozygous variants (>80% variation) among the shared variants of the siblings. An expanded analysis of variants in known inherited retinal disease genes revealed a single heterozygous nonsense variant (c.4723A>T; p.(Lys1575*)) in exon 36 of *CEP290* in both affected persons, which was confirmed by Sanger sequencing. Further analysis of *CEP290* in the exome data did not reveal a second hit, but as coverage of *CEP290* was incomplete, all coding exons and intron–exon boundaries were analyzed by Sanger sequencing. This resulted in the identification of a second nonsense mutation (c.451C>T; p.(Arg151*)) in exon 7 in both siblings which segregated with the disease (Figure 2A). Both mutations were previously described in *CEP290*-related phenotypes with a frequency of 0.00003 (1/28,426 alleles) for c.451C>T and 0.0001 (6/55,820 alleles) In ExAC. 

### 3.3. Analysis of the Hypomorphic Character of c.4723A>T

Compared to the thus far described phenotypes associated with *CEP290* variants, the affected individuals of this family showed a relatively mild non-syndromic retinal phenotype. We therefore hypothesized that the c.4723A>T variant had a hypomorphic character, through a mechanism of nonsense/mediated alternative splicing as was previously experimentally proven for this families’ other *CEP290* allele (c.415C>T) [6]. Reverse-transcription PCR analysis for the latter variant revealed two alternative cDNA products, carrying either a deletion of exon 7 or of exons 7 and 8, both of which are predicted to result in small in-frame (18 or 25 amino acids, respectively) *CEP290* deletions [6]. To determine whether the c.4723A>T variant in exon 36 also induces nonsense-mediated alternative splicing, the cDNA of both siblings was analyzed using a forward primer in exon 34 and a reverse primer in 37. This revealed a weak smaller-sized fragment which lacked exon 36 (Figure 2B,D,F) and results in an in-frame 36-aa deletion (p.(Glu1569_Trp1604del)). This result confirmed the hypothesis of a hypomorphic character of the exon 36 nonsense variant c.4723A>T. The normal-sized 432-bp cDNA products (i.e., containing exon 36) were analyzed by Sanger sequencing. The cDNA obtained from patient EBV cells that were cultured with CHX showed equal amounts of mRNA containing the c.4723A variant (which in fact is the mRNA carrying the c.451C>T variant) and mRNA containing the c.4723A>T mutant allele (Figure 2C), although peak sizes are not directly related to the quantities of the respective mRNA variants. In contrast, the cDNA obtained from patient EBV cells grown without CHX showed more mRNAs containing c.4723A than c.4723T mutant product (Figure 2E), indicating that part of the mRNAs carrying the c.415C>T (p.(Arg151*)) and c.4723A>T (p.(Lys1575*)) variants, underwent NMD. However, one should be cautious interpreting these results as NMD may be different for the RNAs carrying c.4723T and c.451T.

## 4. Discussion and Conclusions

This is the first report on a forty-year follow-up in two siblings initially diagnosed with OT, but converting into a progressive degenerative disease, thereby stressing the importance of studying the natural course of rare diseases. We discovered that the genetic defects were located in *CEP290*, a gene primarily involved in syndromic and non-syndromic LCA, while no pathogenic variants in the coding of near-splice site regions of other currently known inherited retinal disease-associated genes were observed.

OT is a genetically and clinically heterogeneous cone dysfunction syndrome, which is thought to be a stationary condition. A recent OCT study, however, described disease progression based on subtle RPE changes in the perimacular region and focal interruption of the ellipsoid zone on OCT during 18 years of follow up. In this single case, the visual field analysis demonstrated a ring-shaped scotoma around fixation [16]. Andersen et al. and Michaelides et al. reported on patients with OT with subnormal rod-derived ERG responses, a feature also observed in ACHM due to *CNGA3* and *CNGB3* mutations [15,16,23]. We are the first to also describe deterioration of rod-derived responses, associated with progressive peripheral retinal degeneration and increasing visual field loss. 

Although macular changes were observed, individual II-2 still displayed a relatively stable visual acuity and preserved color vision at age 46. In the original report by Van Lith that described the OT phenotype, it was postulated that all three types of cones must be present but reduced in number [11]. His hypothesis was confirmed by Michaelides et al. who used high speed adaptive optics fundus cameras and spectral domain OCT to assess the integrity of the cone photoreceptor mosaic and found that patients with a typical OT phenotype, had a reduced number of cones in the fovea and no obvious cone structure outside the fovea [23]. The reduced cone counts could not explain the complete extinction of the photopic ERG as found in many of the patients, since at least 30% of the normal density was found. 

Applying high-resolution adaptive optics imaging combined with retinal densitometry in healthy individuals, Hofer et al. concluded that the ratio of long-wavelength (L)- and medium-wavelength (M) cones numbers can vary enormously (between 1 and 16), suggesting that relatively normal color discrimination may be preserved, even with the lowest cone number count in the individuals with OT [24]. The findings in subject II-1 of this study with visual evoked potential heterochromatic checkerboard stimulation suggest a protanomaly, which combined with a reduced visual acuity, concentrically reduced visual fields, and nystagmus would indeed make pseudoisochromatic tests difficult, with a preserved Panel D-15 test. 

The OCTs of individual II-2 differ from the ones published by Andersen et al. [13] and Michaelides et al. [23] since our patient had atrophic changes around the fovea and in the posterior pole. In the perimacular region with intact RPE, the three outer retinal layers seemed visible. There was a (double) ring sign observed on FAF in this case indicating the transitional zone between intact and impaired photoreceptor function, a phenomenon also observed in other retinal dystrophies [25,26]. In individual II-1, imaging failed due to the nystagmus and photophobia.

The cone-dominated character of the disease in these two patients is unexpected since gene expression of *CEP290* is seen in the connecting cilia of both rod and cone photoreceptor cells. CEP290 plays a role in primary ciliary assembly [27,28] and has been shown to recruit small GTPase Rab8a (MIM#165040) to the centrosomes and cilia for ciliary membrane elongation [27]. The retinal architecture of *CEP290*-mutant retinas of LCA-patients displayed clear remodeling in the rod-dominated periphery visualized by thickening of the inner retina, whereas the cone-dominated foveal region remained unaltered. Several hypotheses can be put forward for the differences in cone and rod degeneration. First, the difference observed in cone and rod degeneration may indicate different functions of CEP290 in both cell types [29]. As the clinical examinations in our patients uncovered cone defects with late onset progressive peripheral degeneration, one could argue that the cones are more vulnerable compared to rods due to their higher metabolism [30]. The secondary impairment of rods observed in LCA-patients with *CEP290* mutations is also seen in the *Cnga3*^−/−^ mice and *Pde6c^−/−^* zebrafish model, both models for cone degeneration [31,32]. Secondly, it was hypothesized that the cell density plays a role in determining whether there will be secondary rod degeneration. Subsequent to cone degeneration, rod photoreceptor loss occurs in retinal regions characterized by a low density of rods, while the high rod density retinal regions remain intact in mice[32]. A third hypothesis on secondary rod degeneration resides in aberrant gap junction coupling of the photoreceptors [33]. Gap junctions are needed for photoreceptors survival [34] and provide direct electrical coupling between cones and rods [35,36], enabling transmission of signals among the photoreceptor terminals, which subsequently can be transmitted to the inner retina. Whether another hypothesis of damage due to light exposure, as shown for rhodopsin mutations in rodents [37,38], may play a role in this second type of degeneration is speculative. 

Up to now, over 100 different mutations are described in *CEP290*, of which only three are now known to be hypomorphic, but limited genotype–phenotype correlations have thus far been made [9,39]. The hypomorphic deep intronic c.2991+1655A>G variant, resulting in a mixture of truncated (p.(Cys998*)) and wild-type proteins, has been described to cause non-syndromic LCA both in a homozygous state and in a compound heterozygous state together with null alleles [4]. The c.451C>T variant, as described by Littink et al., resulted in a mixture of a predicted severely truncated protein (p.(Arg151*)) and two predicted CEP290 proteins lacking 18 or 25 aa (p.(Leu148_Glu165del) and p.(Leu148_Lys172del)) [6]. The variant reported here (c.4723A>T) gives rise to a major predicted truncated protein (p.(Lys1575*)), and a minor predicted CEP290 protein lacking 38 aa (p.(Glu1569_Trp1604del)). 

The identification of the hypomorphic nature of these three *CEP290* variants warrants the question whether this could be an underappreciated phenomenon in *CEP290-*associated diseases. Eligible mutations that could result in nonsense-mediated altered splicing are located in exons that contain a multiple of three basepairs as their deletion would result in restoration of the open reading frame. Recently, a predictive model based on exon skipping and genetic pleiotropy for *CEP290* mutations was proposed by Drivas et al. In their study, the c.4723A>T variant was considered to have a severe impact that, in a homozygous state, should be associated with Senior Løken syndrome or Meckel–Gruber syndrome [40]. In fact, eight out of nine homozygous cases were diagnosed with LCA (Table 2) [10,41,42,43,44,45,46], which corroborates our findings that the c.4723A>T variant is a moderately severe variant that in a compound heterozygous situation with the hypomorphic variants c.451C>T or 2991+1655A>G results in LCA or OT. Nonsense mutation-mediated exon skipping thereby adds another level of complexity to the interpretation of the effect of sequence variants. 

In summary, we report on two siblings with a rare retinal dystrophy caused by two hypomorphic *CEP290* variants. The identification of hypomorphic variants in *CEP290* raises the possibility that also other ‘milder than presumed’ phenotypes may arise due to *CEP290* mutations. This study also stresses the importance of clinical follow-up of presumably stationary disorders over longer periods of time, to accurately document the disease course. 

## Figures and Tables

**Table 1:** Clinical characteristics of two patients with variants in *CEP290*.

Patient ID	II-1	II-2
Sex	Female	Male
Age at diagnosis	3	2
Age recent examination	46	45
Nystagmus	Present	Present
Visual acuity RE	20/100	20/80
Visual acuity LE	20/100	20/160
Refraction	RE: +1.25–2.25 × 19; LE: −0.25–3.00 × 170	RE: −14.25–1.75 × 5; LE: −10.25–2.25 × 155
Lens	Clear	Clear
Fundus	Pink optic discs, mildly attenuated arterioles, coarse-grained aspect RPE posterior pole and mid-periphery superior quadrants, faintly recognizable foveola reflex BE with subtle indication for bull’s eye-like maculopathy, RPE atrophy in far periphery of superior quadrants, RPE atrophy in mid- and far periphery of inferior quadrants with scarce bone-spicule pigmentations	Pink, myopic optic discs, mildly attenuated arterioles, subtle RPE alterations macula RE, ring-shaped atrophy surrounding the fovea LE, faintly recognizable foveola reflex BE, perimacular RPE atrophy, mild RPE changes superior quadrants, RPE atrophy inferior quadrants with bone-spicule pigmentations
Fundus autofluorescence	Relatively normal	Double hyperautofluorescent ring, hypoautofluorescent spots along and inferior to the inferior vascular arcade BE, and in macula LE
OCT	Failed	No discernible photoreceptor complexes at the macula, but present at the peripheral part of the scan.
Color vision (Panel D-15)	Saturated: normal Desaturated: minor errors RE, multiple errors LE, no specific axis	RE: de- and saturated: multiple errors mainly in tritan axis LE: failed
Visual field (Goldmann)	Radius < 10^0^	Altitudinal defect BE, partially including the center RE, central scotoma LE
ERG Dark adapted	Mildly reduced isolated rod responses with significantly reduced ‘mixed’ responses	Significantly reduced isolated rod and ‘mixed’ responses
ERG Light adapted	Non-recordable	Non-recordable
Miscellaneous	Anorexia, depressions, followed by the diagnosis of schizofrenia at age 29	None

BE = both eyes, ERG = electroretinogram, LE = left eye, OCT = optical coherence tomography, RE = right eye, RPE = retinal pigment epithelium.

**Table 2:** Individuals harboring the c.4723A>T mutation in *CEP290*.

		First Allele			Second Allele			
Sample ID	Diagnosis	DNA Variant	Predicted Protein Variant	Predicted Proteins Based on RNA Study	DNA Variant	Predicted Protein Variant	Predicted Proteins Based on RNA Study	Ref.
*Compound heterozygous*								
Family A	OT	c.4723A>T	p.(Lys1575*)	p.[Lys1575*, Glu1569_Trp1604del] #	c.451C>T	p.(Arg151*)	p.[Arg151*, Leu148_Glu165del, Leu148_Lys172del]	This study
809	LCA	c.4723A>T	p.(Lys1575*)	p.[Lys1575*, Glu1569_Trp1604del]	c.1709C>G	p.(Ser570*)	ND	[10]
LCA-6	LCA	c.4723A>T	p.(Lys1575*)	p.[Lys1575*, Glu1569_Trp1604del]	c.2991+1655A>G	p.(Cys998*)	p.[Cys998*, =] $	[41]
LCA-7	LCA	c.4723A>T	p.(Lys1575*)	p.[Lys1575*, Glu1569_Trp1604del]	c.2991+1655A>G	p.(Cys998*)	p.[Cys998*, =]	[41]
LCA-8	LCA	c.4723A>T	p.(Lys1575*)	p.[Lys1575*, Glu1569_Trp1604del]	c.2991+1655A>G	p.(Cys998*)	p.[Cys998*, =]	[41]
	LCA	c.4723A>T	p.(Lys1575*)	p.[Lys1575*, Glu1569_Trp1604del]	c.2991+1655A>G	p.(Cys998*)	p.[Cys998*, =]	[42]
LCA-24	LCA	c.4723A>T	p.(Lys1575*)	p.[Lys1575*, Glu1569_Trp1604del]	c.4696G>C	p.(Ala1556Pro)	ND	[41]
COR031/CORS1	CORS	c.4723A>T	p.(Lys1575*)	p.[Lys1575*, Glu1569_Trp1604del]	c.4393C>T	p.(Arg1465*)	ND	[10,43]
SLS-2	SLSN	c.4723A>T	p.(Lys1575*)	p.[Lys1575*, Glu1569_Trp1604del]	c.4393C>T	p.(Arg1465*)	ND	[41]
SLS-3	SLSN	c.4723A>T	p.(Lys1575*)	p.[Lys1575*, Glu1569_Trp1604del]	c.4393C>T	p.(Arg1465*)	ND	[41]
F283-21	SLSN	c.4723A>T	p.(Lys1575*)	p.[Lys1575*, Glu1569_Trp1604del]	c.1984C>T	p.(Gln662*)	ND	[44]
A3100-21	SLSN	c.4723A>T	p.(Lys1575*)	p.[Lys1575*, Glu1569_Trp1604del]	c.1987A>T	p.(Lys663*)	ND	[44]
A1210-21	SLSN	c.4723A>T	p.(Lys1575*)	p.[Lys1575*, Glu1569_Trp1604del]	c.3802C>T	p.(Gln1268*)	ND	[44]
F118-21	SLSN	c.4723A>T	p.(Lys1575*)	p.[Lys1575*, Glu1569_Trp1604del]	c.4452_4455delAGAA	p.(Lys1484Asnfs*4)	ND	[44]
A1712-21	SLSN	c.4723A>T	p.(Lys1575*)	p.[Lys1575*, Glu1569_Trp1604del]	c.1189+1A>G	p.(?)	ND	[44]
*Homozygous*								
1	LCA	c.4723A>T	p.(Lys1575*)	p.[Lys1575*, Glu1569_Trp1604del]	c.4723A>T	p.(Lys1575*)	p.[Lys1575*, Glu1569_Trp1604del]	[46]
2	LCA	c.4723A>T	p.(Lys1575*)	p.[Lys1575*, Glu1569_Trp1604del]	c.4723A>T	p.(Lys1575*)	p.[Lys1575*, Glu1569_Trp1604del]	[46]
738	LCA	c.4723A>T	p.(Lys1575*)	p.[Lys1575*, Glu1569_Trp1604del]	c.4723A>T	p.(Lys1575*)	p.[Lys1575*, Glu1569_Trp1604del]	[10]
848	LCA	c.4723A>T	p.(Lys1575*)	p.[Lys1575*, Glu1569_Trp1604del]	c.4723A>T	p.(Lys1575*)	p.[Lys1575*, Glu1569_Trp1604del]	[10]
258	LCA	c.4723A>T	p.(Lys1575*)	p.[Lys1575*, Glu1569_Trp1604del]	c.4723A>T	p.(Lys1575*)	p.[Lys1575*, Glu1569_Trp1604del]	[10]
419	LCA	c.4723A>T	p.(Lys1575*)	p.[Lys1575*, Glu1569_Trp1604del]	c.4723A>T	p.(Lys1575*)	p.[Lys1575*, Glu1569_Trp1604del]	[10]
LEP	LCA	c.4723A>T	p.(Lys1575*)	p.[Lys1575*, Glu1569_Trp1604del]	c.4723A>T	p.(Lys1575*)	p.[Lys1575*, Glu1569_Trp1604del]	[10]
LCA-25	LCA	c.4723A>T	p.(Lys1575*)	p.[Lys1575*, Glu1569_Trp1604del]	c.4723A>T	p.(Lys1575*)	p.[Lys1575*, Glu1569_Trp1604del]	[41]
623	JBTS+retina	c.4723A>T	p.(Lys1575*)	p.[Lys1575*, Glu1569_Trp1604del]	c.4723A>T	p.(Lys1575*)	p.[Lys1575*, Glu1569_Trp1604del]	[10]

Patient LCA-7 and LCA-25 are distantly related; LCA = Leber congenital amaurosis; SLSN = Senior loken; CORS = Cerebello-oculo-renal syndrome; JBTS+retina = Joubert syndrome with retinal involvement; # the mRNA carrying the p.(Lys1575*) mutation is the major product and the mRNA carrying the in-frame amino acid deletion p.(Glu1569_Trp1604del) is the minor product (see Figure 2); ND = not determined; $ = approximately equal amounts of mRNA were detected that contained an intronic insertion resulting in the p.(Cys998*) stop mutation and normal *CEP290* transcript.
